# Prolapsed submucosal pyomyoma postpartum, a rare complication of fibroids

**DOI:** 10.1186/s12884-021-03619-6

**Published:** 2021-02-12

**Authors:** Jingxue Wang, Zheng Li, Yu Sun

**Affiliations:** Department of Obstetrics and Gynecology, Peking University First Hospital, 100034 Beijing, P.R. China

**Keywords:** Pyomyoma, sepsis, Postpartum, Case report

## Abstract

**Background:**

Pyomyoma is an unusual fibroid that usually develops during the puerperal or postmenopausal period. If not promptly diagnosed and treated, it can become life threatening. Although various conservative and surgical therapies have been discussed in the literature for this condition, there are very few reports related to the management of prolapsed pedunculated submucosal myoma.

**Case presentation:**

In this case report, an intramural fibroid transformed into a pedunculated submucosal pyomyoma, which prolapsed into the vagina after a miscarriage and caused life-threatening toxic shock. Apart from prompt antibiotic treatment, a transabdominal myomectomy rather than hysterectomy was performed due to the very large diameter of the pyomyoma. As a result, fertility was preserved, and the patient conceived naturally and delivered a healthy baby two years later.

**Discussion and conclusions:**

It is important to maintain strong clinical suspicion for pregnant or postpartum women with a triad of pain, sepsis without an obvious source and a known diagnosis of leiomyoma. Timely recognition and prompt surgical treatment with antibiotics are necessary and could conserve the uterus for future fertility.

## Background

Pyomyoma, also known as suppurative leiomyoma, is an unusual condition resulting from infarction, necrosis and secondary infection of fibroids [1] that can lead to fatal complications if not promptly diagnosed and treated [2]. It often develops in the puerperal or postmenopausal period [2, 3]; and only twelve cases have been reported during the postpartum period since 1945, but all cases can be accompanied by a suppurative process that develops insidiously [2, 4–6]. Pyomyoma can manifest with a classic triad of symptoms, including abdominal pain, sepsis without any obvious origin of bacteremia and a history of leiomyoma [7]. Hence, high clinical suspicion should be maintained. Management during the postpartum period is complicated and difficult because of its nonspecific performance and a desire to preserve fertility, particularly when pyomyoma occurs after late miscarriage. We present a case of an intramural fibroid that developed during pregnancy and transformed to a pedunculated submucosal pyomyoma and prolapsed to the vagina after miscarriage. The treatment was successful and the uterus was conserved.

## Case presentation

**Fig. 1:**
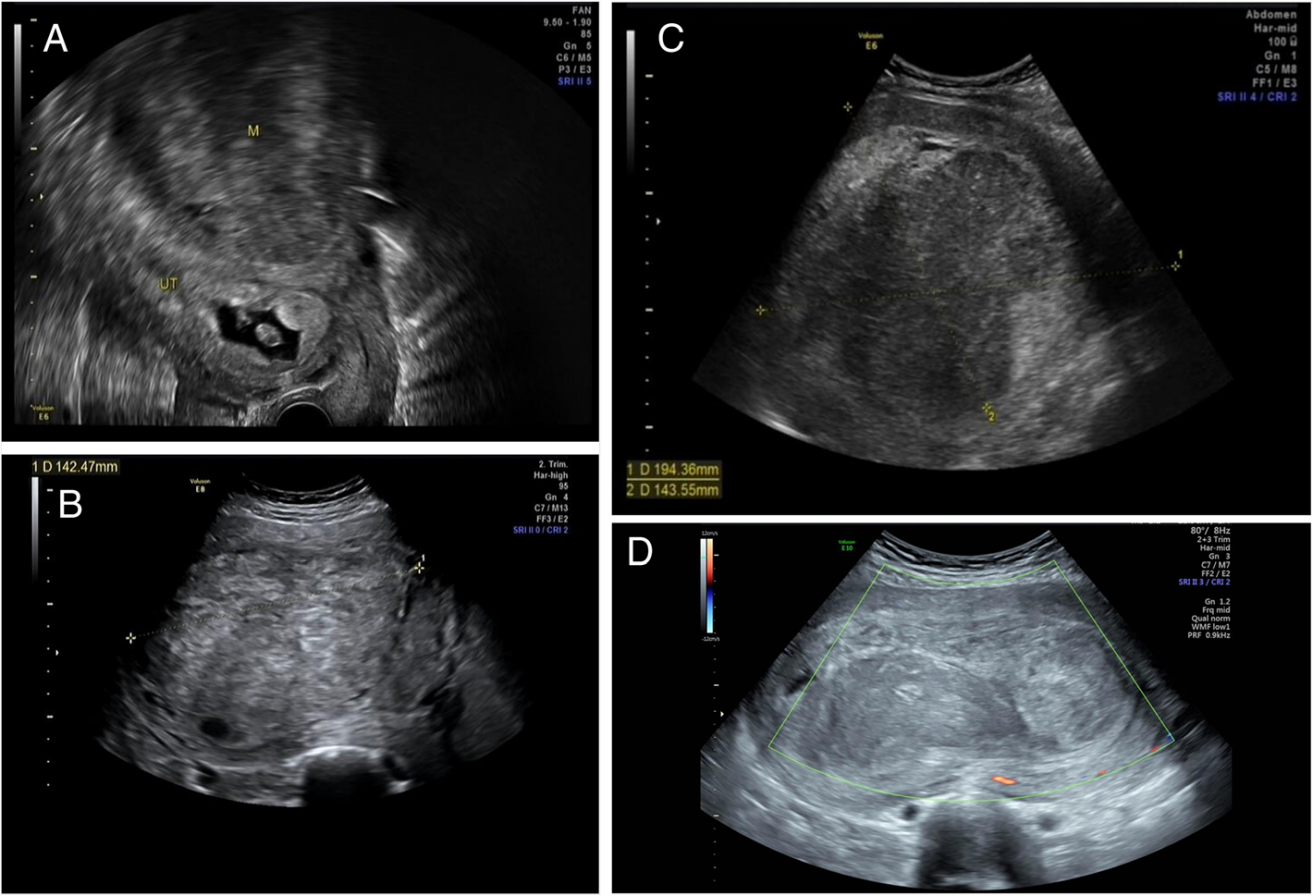
Ultrasound examinations of the fibroids during pregnancy. **a** Ultrasound showing an intrauterine pregnancy and an intramural posterior fundal fibroid; **b** Ultrasound of the original fibroid and two additional fundal fibroids; **c** Large posterior wall fibroid pushing the endometrium anteriorly; **d** Cross-section through the three submucosal myomas. The areas filled with fluid demonstrated a hyperechogenic pattern, which indicated the possibility of degeneration and necrosis

A 24-year-old primigravida was admitted to the Peking University First Hospital at 22^+ 6^ weeks of gestation for a shortened cervical length. Transvaginal ultrasound at the 8th week of gestation revealed a convex, intramural, fundal, posterior wall fibroid measuring 130 mm in diameter (Fig. 1A) with a heterogenic hypoechoic pattern and a local, irregular anechoic area. The fibroid gradually increased in size during the pregnancy. A regular ultrasound at 22^+ 6^ weeks (Fig. 1B) of gestation showed that apart from the initial fibroid, there were two other fundal fibroids with a diameter of 70 mm. The cervical length was 13.2 mm, and the anterior amniotic sac was embedded, wherein flocculent hypoechoic pattern could be detected. A vaginal swab and genital and urine cultures were then taken, which demonstrated negative results. Although the doctors promptly administered expectation treatment, including antibiotics and tocolytics, the patient’s membranes ruptured spontaneously 6 days later, and a lifeless female infant was delivered vaginally. The placenta was fragmented, and the remains were evacuated manually. Repeated genital cultures, including vaginal and cervical cultures and the pathologic results for the placental tissue, were negative, which indicated that there was no evidence of infection, chorioamnionitis or other pathological abnormalities. Over the next nine days, the patient’s swinging pyrexia continued, and the levels of serum markers of inflammation increased. Ultrasonography revealed that the posterior wall fibroid had become larger and the endometrium had pushed anteriorly. The echogenicity of the irregular areas filled with fluid had increased, indicating the possibility of degeneration (Fig. 1 C). The patient was diagnosed with a degenerating fibroid, which may have been the main cause of the persistent fever. Due to the inherent risks of hysterectomy and the desire to preserve fertility, both the team and the patient were eager to avoid surgical intervention unless significant clinical deterioration occurred. The patient was apyrexial after an escalation of antibiotics and was consequently discharged.

Forty days after spontaneous miscarriage, the patient was admitted to the hospital again due to high fever and lower abdominal pain. The patient lost consciousness after a sudden convulsion as soon as she arrived in the emergency room, and she was suspected of having delirium. She demonstrated tachycardia at 173 beats per minute and hypotension at 91/63 mmHg, and her temperature was 39.6°C. A bimanual examination revealed that the uterus was distended up to the umbilicus and was equivalent to 22 weeks in size, and a large, offensive, malodorous, fleshy mass with a friable surface just bulging from the dilated cervix but not protruding into the vagina was found on speculum examination. Immediate ultrasound indicated that the size of the uterus was 16.1 × 7.2 × 21.6 cm^3^, which was consistent with the bimanual exploration. The previously documented intramural fibroids (Fig. 1D) had become submucosal fibroids with diameters of 17, 10, and 9 cm and could be detected in the uterine cavity. The submucosal fibroids had degenerated and necrosed, and a very large heterogeneous echoic pattern bulging into the vagina was detected. C-reactive protein (CRP) was elevated to 43 mg/L, and blood gas analysis revealed respiratory alkalosis. Aerobic and anaerobic blood culture results suggested *Klebsiella pneumoniae* infection, and the cultures showed negative results. The patient was started on meropenem for presumed ongoing sepsis. Resuscitation measures, including quick fluid rehydration, were taken.

**Fig. 2:**
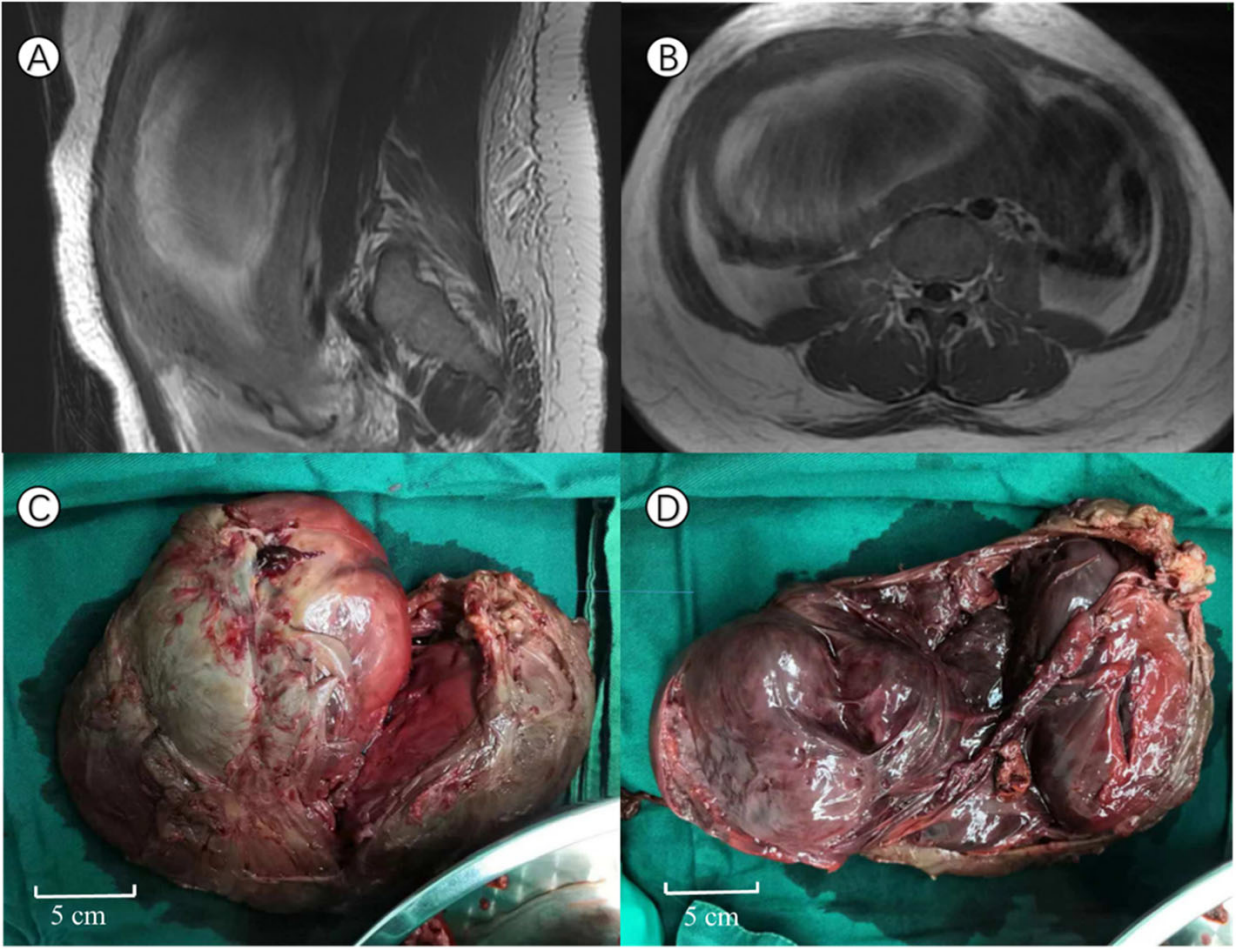
MRI examinations and grossspecimens of the submucous pyomyoma postpartum. **a **and **b** Coronal and horizontal MRI showing abnormal signals in the uterine cavity, suggestive of necrotic tissue. **c** and **d** Fibroid after removal

The following day, serum blood tests showed a procalcitonin level of 41 ng/mL, a white blood cell count of 4.16 × 10^9^/L and a CRP level increased to 54 mg/L. The patient showed no significant improvement in symptoms and presented with edema of the limbs and face, diarrhea, abnormal blood coagulation function and multiple organ dysfunction. The diagnosis of sepsis was doubtless, and vancomycin was added to her antibiotic regimen. A computed tomography (CT) scan of the abdomen and magnetic resonance imaging (MRI) of the pelvis (Fig. 2A and B) were performed to search for other intra-abdominal sources of the sepsis. The examinations revealed a 13 cm submucosal fibroid with degeneration and necrosis and dilatation of the maternal renal pelvis.

The decision was made to correct the sepsis and remove the fibroids at the same time if possible. Informed consent for the operation covering all possible risks was signed. Transvaginal mass resection was performed first, and approximately 200 mL of dark red blood clots and necrotic membrane-like tissue were removed. However, it was rather difficult to clamp the whole fibroid. Thus, exploratory laparotomy was performed, and it was revealed that a large, foul, malodorous submucosal fibroid had filled the whole uterine cavity, which had been distended to up to 20 weeks in size. The fibroid was attached at the fundus with a relatively short but broad band and was completely enucleated. The specimen measured 20 × 15 × 10 cm^3^ and weighed 650.0 g (Fig. 2C, 2D). Postoperative incision of the myoma indicated a solid mass composed of uniform and soft tissue with a central area of cystic degeneration. Histopathologic examination confirmed that the lesion had a bacterial structure, and neutrophilic infiltration in the focal point was the portion of the fibroid that demonstrated significant degeneration and suppurative necrosis. However, the results of intraoperative cultures were negative.

Postoperatively, the patient was given seven days of meropenem and vancomycin intravenously, resulting in a good clinical response and infection marker levels. She was discharged on the seventh day after surgery and was required to take meropenem orally for 7 days. At the 2-, 5- and 7-month follow-up visits, the patient remained stable and symptom free. She conceived naturally and delivered a live, healthy, full-term baby by cesarean section two years later.

## Discussion and conclusions

Herein, the authors presented a rare and serious complication of fibroids, pyomyoma, which has been described as a life-threatening infection of fibroids with an incidence of approximately 0.5 % before the application of antibiotics [8–10]. It may arise as a consequence of uterine manipulation, such as abortion and cesarean section. Only 12 cases occurring during the postpartum period have been reported since 1945, and most infections in the postpartum period developed insidiously over days to weeks between delivery and the initial onset of symptoms [11]. As described in this case, pyomyoma may be clinically silent despite producing continuous bacteremia and is difficult to diagnose early during the subacute course. The transmission route postpartum is usually ascending infection, which will lead to the colonization of necrotic tissue. However, it can also spread from the uterine cavity or any adjacent structures or by distant hematogenous/lymphatic spread [4, 11–14].

Although no specific pathogen was found in this case, the second trimester miscarriage and persistent fever postpartum indicated potential infection. Interestingly and uniquely, in this case, the intramural fibroids seen in the first trimester relocated to a pedunculated submucosal form 40 days after parturition, which increased the chance of ascending infection. It is likely that the migration of the fibroid had taken place over an extended period of time. This phenomenon of fibroid relocation during pregnancy has been reported in very few case reports, and the mechanism and etiology underlying this relocation are currently unexplained [15, 16]. Wael Elgonaid described a case involving a large prolapsed submucosal postpartum myoma, which was originally a known hybrid, predominantly intramural myoma and caused an infection similar to that observed in this case [17]. Pedunculated submucosal myoma may be prone to necrosis due to the lack of an adequate blood supply through the pedicle. From a management perspective, the subgroup of prolapsed pedicled submucosal fibroids should be treated differently from other types of uterine fibroids because of their diagnostic and management difficulty [18].

Unlike the submucosal pyomyoma, which obviously prolapse, fever is generally the only symptom in some reported cases, especially in the early stages, which may be misleading and delay the initiation of treatment. Moreover, the patient may present with complaints of abdominal pain or an abdominal mass. Sometimes an acute abdomen may occur, which is potentially due to serious complications such as a ruptured pyomyoma [19], peritonitis and sepsis. Thus, the combination of known fibroids with sepsis in the absence of other sources of infection for bacteremia should lead to a strong clinical acumen and a high index of suspicion of pyomyoma [5, 7]. The differential diagnosis of pyomyoma includes any pelvic mass associated with signs of infection (pyometra, tuboovarian abscess), malignancy, degenerated leiomyoma, and ectopic infection [20].

It is recommended that treatment should be administered after all the relevant preoperative investigations, including the following imaging modalities, have been performed. Ultrasound scans could detect the position and size of the pyomyoma, but these may be insufficiently distinct for diagnosis. CT and MRI, which can show large heterogeneous echogenic pelvic patterns with solid and cystic components correlated with areas of degeneration and infarction, contribute to an early definitive diagnosis [21, 22].

If pyomyoma is highly suspected during pregnancy and in the postpartum period, the signs of infection and ultrasound examination should be monitored closely and regularly. For pregnant women with pyomyoma, the gestational week should be extended as long as possible if the clinical condition is stable. However, if there are signs of infection or other potential serious complications, termination of the pregnancy should be discussed according to the actual condition. During puerperium, scheduled or emergency surgical treatment with powerful anti-inflammatory effects should be selected. In addition, it is vital to differentiate the retained products of conception from the sloughed necrotic submucosal myoma through clinical examination and relevant imaging modalities and histology when the patient presents fever and tachycardia after delivery or miscarriage.

Although pyomyoma may lead to adverse obstetric outcomes, prompt surgical intervention combined with appropriate antibiotic treatment can improve the clinical prognosis and prevent fatal complications and has therefore become the primary and definitive management, as described in most reported cases [7, 23]. Surgery generally involves myomectomy and hysterectomy with an open approach. Myomectomy can even be safely performed during the first and second trimesters of pregnancy and allow fertility preservation [11, 23]. There are still no guidelines on the management of prolapsed pedunculated submucosal pyomyoma, which can be removed transabdominally or vaginally, as corresponding data on these two approaches are very limited. For the patient in this case, considering the large diameter of the pyomyoma and multiple fibroids (more than 15 cm), we performed transabdominal myomectomy after an attempted vaginal myomectomy under ultrasound guidance. For postmenopausal women, total abdominal hysterectomy is a preferred option, which will lead to irreversible fertility. Nevertheless, a more conservative approach, CT-guided drainage and lavage of pyomyoma, has been reported recently [5, 24]. This is a minimally invasive alternative for nulli- and primiparous women and could resolve the clinical symptoms of infection and reduce the risks of extensive surgery and should be explored in future cases. However, the rate of cure and recurrence has not been reported, and surgical intervention remains the treatment of choice if unsuccessful.

In summary, this case depicted a fibroid during pregnancy whose anatomical site changed from intramural to submucosal type and that prolapsed and became infected postpartum. Timely recognition and treatment of pyomyoma improve the clinical results. Pyomyoma should be differentially diagnosed for any patient with pyrexia and abdominal pain and should be extremely suspected if the patient has a history of leiomyoma. Prompt surgical treatment with antibiotics is necessary and can conserve the uterus for future fertility.

## Data Availability

The datasets used and/or analysed during the current study available from the corresponding author on reasonable request.

## Abbreviations

CRP: C reactive protein
CT: Computed tomography
MRI: Magnetic resonance imaging
